# P16 UTI Friday: a review of antibiotic prophylaxis in the management of recurrent UTI in primary care

**DOI:** 10.1093/jacamr/dlad066.020

**Published:** 2023-06-14

**Authors:** Avril Tucker

**Affiliations:** Cwm Taf Morgannwg University Health Board, UK

## Abstract

**Background:**

An appropriate and evidence-based strategy for treating patients with recurrent urinary tract infection (rUTI) is to use low-dose antibiotics as prophylaxis. All antibiotics used for rUTI prophylaxis are associated with problems of resistance and adverse effects. Antimicrobial resistance (AMR) refers to a natural phenomenon in which micro-organisms become resistant to antibiotics that were originally effective in killing them. There is an established link between antibiotic use and the emergence of resistant organisms. Current NICE guidelines (NG15, NG112) state that patients on antibiotic prophylaxis for rUTI should have a clinical review within 6 months to assess success from the prophylactic regimen. At this review, consideration should be given to discontinuing the antibiotic and the promotion of self-care measures in its place. This project aims to assess current practice in relation to rUTI management in primary care and identify opportunities for improved antimicrobial stewardship.

**Methods:**

An audit was developed to review rUTI management, and it was used in three GP practices (patient population 35 134) within the Bridgend Locality of Cwm Taf Morgannwg University Health Board. If regular antibiotic prophylaxis is required, nitrofurantoin 50 mg/100 mg nocte or trimethoprim 100 mg nocte should be considered first line, unless associated with resistance, allergy or co-morbidities. Cefalexin 125 mg nocte is the second choice. A search for all repeat prescriptions issued for any strength of these drugs, from 1 July 2017 to 30 June 2018, was run in the GP practice systems, and 179 patients were identified as being on antibiotic prophylaxis for rUTI (∼0.5% of the practice populations). The results of the audit were fed back to the practice, and a co-designed action plan agreed to review the identified patients. The outcome of this activity was the proof of concept for national roll-out and lead to the audit being included in a ‘basket’ of Quality Improvement Projects as part of the Welsh General Medical Services (GMS) 2019/20 contract. Due to the COVID pandemic, it remained live until September 2022.

**Results:**

Primary care–initiated prophylaxis accounted for 69% of the total number of patients identified (*n* = 123). Of those taking prophylaxis for >6 months (*n* = 160), 24% had a prophylactic review within the previous 6 months as per NICE guidance. Patients taking prophylaxis for >3 years accounted for 39% of the total (*n* = 70). Whilst taking antibiotic prophylaxis, 98 patients (54.7%) had at least one documented acute UTI in the previous 12 months. For an acute UTI, 35 patients (19.6%) were prescribed the same antibiotic as they were taking prophylactically. When an MSU showed resistance to the prophylactic antibiotic, prophylaxis was changed 17.5% of the time, *n* = 57. Therefore, 82.5% were continued on a potentially ineffective antibiotic. Results from the Welsh GMS contract for the Bridgend locality (patient population 152 041) identified 431 patients on antibiotic prophylaxis for rUTI. Of these, 334 (77.5%) were reviewed, and of those reviewed, 171 (51%) were stopped (confirmed on re-audit after 6 months). This equates to a 40% locality reduction in antibiotic prophylaxis for rUTI, which is estimated to be in the region of 2223 fewer antibiotic prescriptions per year.

**Conclusions:**

Initial results from the *UTI Friday* audit suggest that there can be marked improvement in antibiotic use for rUTI management. Through a multi-disciplinary approach, this project has demonstrated how pharmacists can support GPs in the review and management of patients with rUTI. Using audit feedback, co-designed action plans have been implemented. The impact of this on resistance seen in urinary isolates is not yet known but, with increased guideline awareness/compliance, a positive step has been made towards improving rUTI management and meeting the antimicrobial stewardship targets set by the UK Antimicrobial Resistance National Action Plan 2019–2024. In order to tackle high rates of antibiotic prescribing in primary care, innovative approaches to quality improvement and prescriber feedback will be essential and should be further developed.

